# Impact of social relationships on Alzheimer’s memory impairment: mechanistic studies

**DOI:** 10.1186/s12929-018-0404-x

**Published:** 2018-01-11

**Authors:** Ya-Hsin Hsiao, Chih-Hua Chang, Po-Wu Gean

**Affiliations:** Department of Pharmacology, College of Medicine, National Cheng Kung University, No.1, Ta-Shieh Rd, Tainan City, 701 Taiwan

**Keywords:** Alzheimer’s disease, Social isolation, Hippocampus, Cognition, Epigenetic, BDNF, Neurogenesis

## Abstract

Alzheimer’s disease (AD) is characterized by progressive memory and neuronal loss culminating in cognitive impairment that not only affects a person’s living ability but also becomes a society’s as well as a family’s economic burden. AD is the most common form of dementia in older persons. It is expected that the number of people with AD dementia will increase dramatically in the next 30 years, projecting to 75 million in 2030 and 131.5 million in 2050 worldwide. So far, no sufficient evidence is available to support that any medicine is able to prevent or reverse the progression of the disease. Early studies have shown that social environment, particularly social relationships, can affect one’s behavior and mental health. A study analyzing the correlation between loneliness and risk of developing AD revealed that lonely persons had higher risk of AD compared with persons who were not lonely. On the other hand, it has been reported that we can prevent cognitive decline and delay the onset of AD if we keep mentally active and frequently participate in social activities. In this review, we focus on the impact of social behaviors on the progression of cognitive deficit in animal models of AD with a particular emphasis on a mechanistic scheme that explains how social isolation exacerbates cognitive impairment and how social interaction with conspecifics rescues AD patients’ memory deficit.

## Background

An early sign of Alzheimer’s disease (AD) is frequent forget of recently happened events, which culminates in cognitive impairment that not only affects a person’s living ability but also becomes a society’s as well as a family’s economic burden. AD is the most common form of dementia in aged persons [1–3]. It was estimated that there were 5 million individuals with AD dementia in 2010 in the USA. The number will increase dramatically in the next 30 years, projecting to be 16 million according to the Alzheimer’s association report (2017). In histopathological examination, AD is typically characterized by two biomarkers: neuritic plaques consisting of amyloid beta (Aβ) peptide, and neurofibrillary tangles consisting of hyperphosphorylated tau protein [4, 5]. Accumulating evidence indicates that plaques and neurofibrillary tangles are caused by glial activation, neuritic dystrophy and loss of synapses, and neuron death [6]. Currently, no particular medicine has been definitely shown to be able to prevent or reverse the progression of AD [7]. Previous studies suggest that the brain levels of Aβ are correlated with and may be the cause of memory deficit in patients developing AD [8, 9]. The amyloid precursor protein (APP) is an integral membrane protein that the Aβ is derived from. [10]. APP can be cleaved by β-secretase and γ-secretase at the N and C termini to yield Aβ, which is released into the extracellular space [11, 12]. The precise mechanisms by which Aβ impairs synaptic plasticity and induces memory deficit are not fully understood. Aβ-induced endocytosis of synaptic NMDA receptors [13, 14] and AMPA receptors [15], decrease in dendritic spines and disruption of the cytoskeletal network [16], activation of protein (STEP) [17–19], and disruption of neuronal glutamate uptake [20] in the affected brain regions could be the underlying mechanisms.

Mild cognitive impairment (MCI) causes a slight decline in cognitive abilities but the decline is not sufficient to interfere with daily activities [21]. People with MCI are more likely to develop AD or other dementias at a rate of approximately 10% to 15% per year [22, 23]. However, MCI does not always progress to dementia. In clinical trials, it has been repeatedly demonstrated that cognitive function can be improved in elders including AD patients after physical exercise training [24, 25]. In contrast, medical conditions such as high blood pressure, hypercholesterol, mental illness, and lifestyle factors like infrequent physical exercise and seldom social activity may aggravate cognitive decline. It is thus advised that people seek help from medical doctor for diagnosis and possible treatment when they begin to experience difficulty in recollection. In this review, we focus mainly on the impact of social behaviors on the progression of cognitive impairment in the animal model of AD.

### Factors that regulate Alzheimer patients’ cognitive impairment

Early studies have shown that social environment, particularly social relationships, can affect one’s behavior and mental health [26]. A longitudinal cohort study analyzing the correlation between loneliness and the risk of developing AD revealed that persons who were living lonely had higher risk of AD compared with persons who were not [27]. Unexpectedly, brain autopsy showed that loneliness score was not correlated with Aβ-immunoreactive plaques and cerebral infarction, arguing against the direct contribution of social isolation to the risk of AD and the underlying mechanism is unrelated to AD pathology [27]. In contrast, a later study using the Pittsburgh Compound B (PiB)-PET criteria to measure fibrillary amyloid burden revealed a correlation between loneliness and elevated cortical amyloid in cognitively normal older adults, suggesting that loneliness is a risk factor relevant to preclinical AD [28]. Thus, the neurobiological mechanisms underlying memory decline associated with social isolation remain elusive.

Social isolation, the absence of interaction with others, is considered the major source of mental and psychosocial stress, which contributes to the increased prevalence of neurological diseases [29]. It also increases the risk of morbidity and mortality as well as the onset of many neuropsychological disorders [29–32]. In rodents, it has been repeatedly demonstrated that social isolation exacerbates memory deficit in the animal model of AD [33, 34]. The mechanisms mediating this impairment are not yet completely understood but may include the production of Aβ peptide and the phosphorylation of tau protein [35, 36], an increase in oxidative stress and inflammatory reaction [37] accompanied by inhibition of anti-inflammatory responses [38], synaptic plasticity including the reduction of brain-derived neurotrophic factors (BDNF) [39, 40], and myelination [41]. Social isolation may also decrease hippocampal BDNF gene H3 acetylation and BDNF protein expression [42]. Consistently, social isolation-induced anxiety- and autism-like behaviors are lessened by hippocampal BDNF over-expression [43]. However, contrast results have been reported in the female mouse brain in which social isolation-induced anxiety-like behavior is mediated by the upregulation of mRNA and protein levels of BDNF in the cerebral cortex [44]. Sex difference in response to stress or brain region difference (cerebral cortex vs. hippocampus) may account for the differential results. Finally, social isolation-induced aggression-related disorders are reversed by combined treatment with antidepressant fluoxetine plus re-socialization that is able to restore BDNF expression through epigenetic regulation [45].

**Fig. 1:**
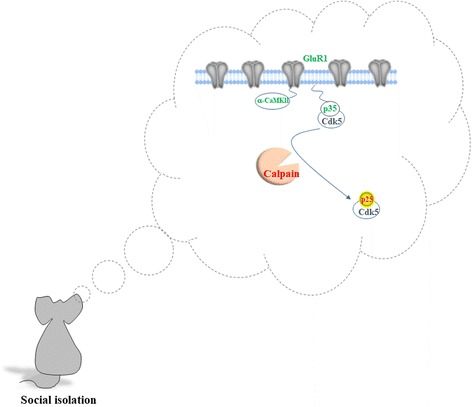
Schematic diagram illustrates how social isolation exacerbates memory decline in APP/PS1 mice. We propose that social isolation increases calpain activity leading to conversion of p35 to p25. Decrease in membrane associated p35 reduces α-CaMKII synaptic distribution resulting in the synaptic removal of AMPARs

We have studied the cellular mechanisms of how social isolation aggravates AD-associated memory decline in APP/PS1 mice. We first constructed age-dependent memory performance using a contextual fear conditioning paradigm and demonstrated that the memory of APP/PS1 mice is normal at 3 months of age and begins to declines at 6 months. However, APP/PS1 mice reared in socially isolated cages exhibited memory deficit as early as 3 months of age, which was associated with increases in hippocampal Aβ, calpain activity, and p25/p35 ratio concomitant with decreases in membrane-associated p35, GluR1, and GluR1 Ser831 phosphorylation, and surface α-amino-3-hydroxy-5-methyl-4-isoxazolepropionic acid receptors (AMPARs) [36]. We conducted immunoprecipitation experiments using antibody against p35 and found that α-CaMKII, GluR1, and PSD-95 were associated with p35. Reciprocal immunoprecipitations using antibodies against α-CaMKII, GluR1, and PSD-95 confirmed these molecules were associated with p35. Thus, it is proposed that social isolation facilitates memory decline by increasing the Aβ level and calpain activity, subsequently leading to the conversion of p35 to p25 and decreased association of p35, α-CaMKII, and GluR1, resulting in the endocytosis of AMPA receptors (Fig. 1).

Exercise regularly and eat a healthy diet rich in fruits and vegetables are known to be beneficial for preventing cognitive decline with aging and AD. Exercise may induce biochemical changes in the brain areas important for learning and memory such as hippocampus and related medial temporal lobe circuitry [46]. Current evidence from multi-country ecological and observational studies suggests that the traditional Mediterranean diet is a model of healthy eating in terms of the reduced incidence of cardiovascular and chronic degenerative diseases. The Mediterranean diet is composed of abundant fruits, vegetables, breads, other forms of cereals, potatoes, beans, nuts, and seeds. In contrast, the Western dietary pattern, with large amounts of meat, is highly correlated with the risk of developing AD [47, 48].

A previous study showed that social stress activated the sympathetic nervous system and increased bone marrow production of immature proinflammatory monocytes, which could be blocked by β-adrenoreceptor antagonists [37]. Social isolation also increased superoxide-producing nicotinamide adenosine dinucleotide phosphate (NADPH) oxidase 2 (NOX2) expression and immunoreactive microglia [9]. Social isolation-induced behavioral and histopathological changes were ameliorated by administration of antioxidant/NOX inhibitor apocynin, suggesting that increased oxidative stress underlay the adverse effects of social isolation [34, 49, 50]. Consistent with these results, in APP/PS1 mice, we found that γ-secretase activity and levels of Aβ-40 and Aβ-42 were elevated in socially isolated mice compared to those of mice in group housing. N-acetylcysteine (NAC) is a potent antioxidant which has been demonstrated to protect the brain from focal cerebral ischemia [50]. Chronic treatment of mice with NAC reversed the elevated γ-secretase activity and Aβ-40 and Aβ-42 levels in isolated APP/PS1 mice [51]. It is likely that NAC’s antioxidant effect counteracts social isolation-induced oxidative stress (50), increased γ-secretase activity and Aβ levels [52, 53].

### Social interaction rescues Alzheimer patients’ memory deficit by increasing BDNF expression

BDNF produces many beneficial effects on brain functions. For example, it increases synaptic plasticity and enhances neurogenesis and cognitive functions [54]. In contrast, decreased BDNF and its receptor tropomyosin-related kinase B (TrkB) contribute to cognitive decline in aging [55, 56]. The levels of serum BDNF in patients with drug-naïve first episode depressive disorder were significantly lower as compared to healthy controls [57]. Reduced protein and mRNA levels of BDNF were found in the hippocampus of postmortem AD samples and MCI patients, [58–60]. In aged rats and primates, BDNF infusion produced beneficial effects on age-related perturbations in gene expression and ameliorated age-related cognitive impairment [61]. Furthermore, systemic administration of a small-molecule TrkB agonist, 7,8-dihydroxyflavone (7,8-DHF), in amyloid precursor protein (APP) transgenic mice improved their synaptic loss and memory deficit [62–65].

Clinicians and medical reports often advise us that one can reduce the risk of cognitive rundown and delay the onset of AD if one maintains strong social connections [66, 67]. Indeed, a relationship between frequent social activities and better cognitive function has been established [66–68]. However, the mechanisms behind social and emotional influence are largely unknown. We investigated the hypothesis that having a company may produce a beneficial effect on the cognitive function in AD mice. Indeed, the results showed that APP/PS1 mice performed better in memory tasks if their companies interacted with them more often.

**Fig. 2:**
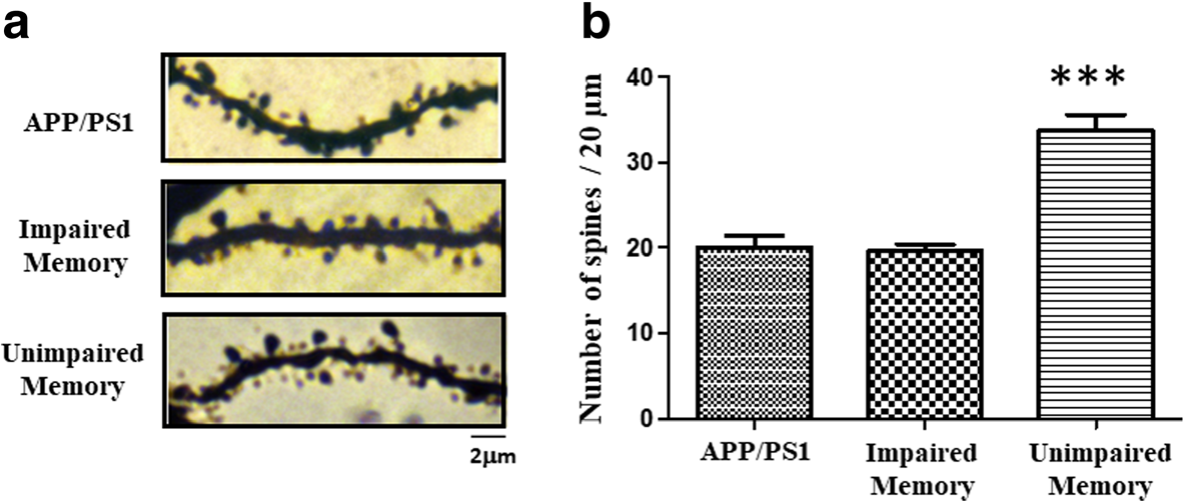
Co-housing with conspecific increases spine density in hippocampal CA1 neurons of the AD mice. Representative images (**a**) and statistical analyses of Golgi staining (**b**) from the hippocampal CA1 neurons of the control, memory-unimproved and memory-improved AD mice after co-housing with conspecific. ***p < 0.001 vs. memory-unimproved

Experience-induced changes in dendritic spine stability serve as the mechanism for the maintenance of long term memories. We found that co-housing-induced improvement of memory in the AD mice was accompanied by an increase in spine density in the hippocampus (Fig. 2). Furthermore, we demonstrated that the beneficial effect of co-housing was mediated by increased BDNF expression and subsequent neurogenesis in the dentate gyrus (DG) of the hippocampus [65].

These results are consistent with recent cohort studies. Among 3294 participants controlled for age and sex, Salinas et al. found that participants with more companionship had higher serum BDNF level and reduced risk for dementia [69]. Similar results were observed in rodents that had been reared in a communal nest (CN), a form of early social enrichment. These mice showed higher BDNF levels in the brain [70].

It is known that hippocampal BDNF modulates neurogenesis, synaptic plasticity, and behaviors in rodents [71–73]. Hippocampal neurogenesis can be regulated by environmental factors such as environmental enrichment and exercise [74]. For example, exercise improves social isolation-induced impairment of cognitive performance and reduction of hippocampal BDNF [75]. Rodents housing with a running wheel over a six-month period [76] and exercise [77] can partially reversed age-dependent reduction in neurogenesis and cognition.

To investigate whether neurogenesis in the hippocampus plays a critical role in cohousing-induced reversal of cognitive decline in APP/PS1 mice, the cell proliferation blocker methylazoxymethanol acetate (MAM) was used [78]. We found that blocking neurogenesis in the hippocampal DG prevented cohousing-induced rescue of memory impairment in APP/PS1 mice [65].

We further investigated the role of newborn neurons in cohousing-induced reversal of memory decline. A retrovirus vector encoding diphtheriatoxin receptor (DTR) was injected into the dentate gyrus to tag and target mitotic neurons for subsequent ablation [79, 80]. When diphtheriatoxin (DT) was bound to the receptor, mitotic neurons with DTR underwent apoptotic cell death. Administration of DT significantly reduced BrdU^+^/NeuN^+^ cells in the DG and the improvement of memory after cohousing was abolished. These results suggest that neurogenesis in the hippocampal DG is required for the rescue of memory impairment in APP/PS1 mice.

### Epigenetic regulation of BDNF expression by social interaction

Epigenetic mechanisms refer to regulatory modifications of gene expression without changing gene sequence [81–83]. Such effects provide an organism with the molecular mechanisms to promptly adapt to environmental changes as well as to regulate diverse biological processes [84] with stable alterations in gene expression. Epigenetic modifications are commonly regulated by chromatin remodeling via histone acetylation, or by direct methylation of DNA. Chromatin remodeling through histone-tail acetylation alters the compact chromatin structure and enables the transcriptional machinery to access the transcriptional start site [85, 86]. Conversely, histone deacetylases (HDACs) repress transcription by condensing the chromatin via removing acetyl groups from core histone proteins. HDAC proteins are classified into four classes based on three-dimensional structures, substrate specificity and DNA sequence homology to the yeast original enzymes. Classes I, II, and IV belong to classical HDACs and their activities are inhibited by trichostatin A (TSA), whereas Class III HDACs are a family of NAD^+^-dependent proteins having sequence similarity to the yeast Sir2 protein [87]. HDAC2, a Class I HDAC, is localized to the promoters of numerous synaptic plasticity-associated genes, including *Bdnf* promotor IV, where it deacetylates histone substrates and negatively regulates gene transcription [88, 89]. Consequently, knockdown of HDAC2 or treatment with HDAC inhibitor promotes synaptic gene expression, long-term synaptic plasticity, and memory retention [88–91].

Voluntary wheel running and environmental enrichment have been shown to increase BDNF gene expression in the hippocampus of rodents [92–97]. The mouse BDNF gene contains multiple 5′ noncoding exons and a single 3′ coding exon (exon IX) for the BDNF protein [98]. The noncoding exons undergo alternative splicing and join exon IX to produce multiple exon-specific BDNF transcripts for fine-tuning transcription in different cell types and by different neuronal activities. Previous studies have shown that exercise stimulates DNA demethylation by inducing methyl-CpG-binding protein 2 (MeCP2) phosphorylation resulting in the dissociation of phospho-MeCP2 from the *Bdnf* promoter, and leading to *Bdnf* transcription in the rat hippocampus [99]. In addition, the chromatin immunoprecipitation assay revealed that exercise increased histone H3 acetylation and reduced mRNA and protein levels of HDAC5. Similarly, a marked increase in the BDNF mRNA in the hippocampus was observed in mice housed in an enriched environment for 3–4 weeks. Correspondingly, the enriched environment reduced histone H3 lysine 9 (H3K9) trimethylation at the BDNF P4 promoter and histone H3 lysine 27 (H3K27) trimethylation at the BDNF P3 and P4 promoters, leaving histone methylases and demethylases expression in the hippocampus untouched [100].

**Fig. 3:**
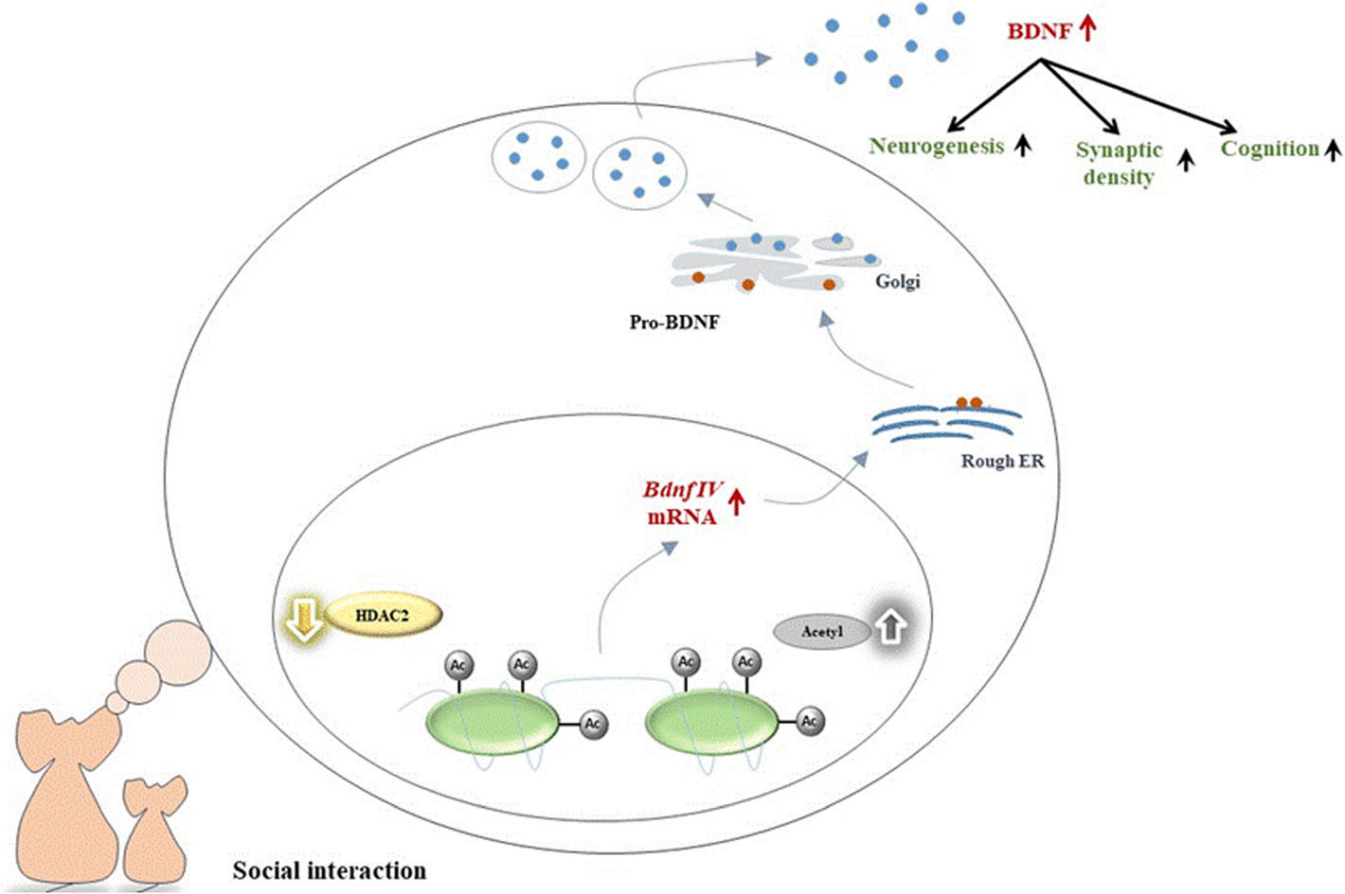
Schematic diagram illustrates how co-housing reverses memory decline in APP/PS1 mice. We propose that co-housing reduces HDAC2 expression and the occupancy of HDAC2 in the promoter region of *Bdnf* exon IV resulting in the increased levels of acetylated histone H3K9 and H4K12. This leads to the higher transcription and translation of BDNF mRNA and protein in the hippocampus that improves memory. (Modified from reference [65])

Postmortem studies reported that BDNF expression is lower in AD patients [57, 101]. Infusion of amyloid fibrils significantly increased HDAC2 expression and its occupancy in the promoter region of *Bdnf* exon IV, resulting in histone H3 deacetylation and the suppression of BDNF expression [102, 103]. We examined epigenetic changes in the mouse hippocampus that accompanied cohousing-induced memory improvement. We found that the level of HDAC2, but not that of HDAC1, was significantly lower in the memory-improved mice than in the control and memory-unimproved APP/PS1 mice after cohousing. Knockdown of *Hdac2* resulted in a higher freezing response after cohousing. Conversely, over-expression of HDAC2 blocked cohousing-induced memory improvement. The level of *Bdnf* exon IV mRNA increased significantly after knockdown of *Hdac2*. The ChIP assay revealed a decreased occupancy of HDAC2 in the promoter region of *Bdnf* exon IV of memory-improved mice but not memory-unimproved and control APP/PS1 mice. Consistently, the acetylation of histone 3 on Lys 9 (H3K9) and histone 4 on Lys12 (H4K12) increased significantly in the promoter region of *Bdnf* exon IV [104]. These results suggest that HDAC2 expression is reduced after cohousing that results in a decreased occupancy of HDAC2 and increased histone H3K9 and H4K12 acetylation in the promoter region of *Bdnf* exon IV. This leads to increased BDNF expression in the hippocampus and improves memory (Fig. 3). Further support for this notion comes from a study by Yamakawa et al., who investigate proteins that mediate HDAC2 recruitment to synaptic plasticity genes using an integrative genomics approach [105]. They have demonstrated that Sp3 and HDAC2 interacts to repress gene expression and negatively regulates synaptic plasticity.

It is noted that, besides BDNF, the neurotrophin family also comprises nerve growth factor (NGF), neurotrophin 3 (NT3), and neurotrophin 4/5 (NT4/5) [106]. In nucleus basalis of Meynert, decreased NGF level has been shown to be associated with neurodegeneration of AD and treatment with NGF improved cognitive performance [107]. In addition, NGF gene therapy trials using NGF-grafted autologous fibroblasts increased neuronal responses in AD patients [108]. Therefore, epigenetic regulation of NGF expression by social interaction warrants further investigation.

## Conclusion

Accumulating evidence suggests that both humans and mice have a higher risk of developing AD if they are lonely or living isolated. One mechanism can be accounted by the social stress-induced increased Aβ and calpain activity which leads to conversion of p35 to p25 and decreased association of p35, α-CaMKII, and GluR1, resulting in the removal of AMPA receptors from synaptic membrane. In contrast, AD mice can improve cognitive function if they are accompanied and interacted with conspecifics. Reduction in the level of HDAC2 as well as decreased occupancy of HDAC2 in the promoter region of *Bdnf* exon IV is likely the underlying mechanism. Consistently, HDAC inhibitor treatments and direct HDAC2 knockdown are able to recover impaired cognitive functions in the AD mice. Thus, targeting HDAC2 or inhibiting HDAC2-Sp3 binding can be a potential strategy against AD development.
